# Effective debriefings in the clinical setting: a pilot study to test the impact of an evidence based debriefing app on anesthesia care providers’ performance

**DOI:** 10.3389/fmed.2024.1427061

**Published:** 2024-07-26

**Authors:** Julia C Seelandt, Jeannine Schneider, Michaela Kolbe, Bastian Grande

**Affiliations:** ^1^Simulation Center, University Hospital Zurich, Zurich, Switzerland; ^2^Institute of Anesthesiology, University Hospital Zurich, Zurich, Switzerland; ^3^Department of Health Sciences and Technology, ETH Zürich, Zürich, Switzerland

**Keywords:** self-debriefing, reflective statements, team learning, team performance, debriefing application

## Abstract

**Background:**

Debriefing enhances team learning, performance, and patient safety. Despite its benefits, it’s underused. To address this, we developed an evidence-based debriefing app.

**Methods:**

This pilot study, conducted at a Swiss hospital, evaluated team performance during two anesthesia inductions using the Team Performance Scale (TPS). Following the first induction, teams engaged with the Zurich Debriefing App, with debriefing sessions meticulously recorded for subsequent evaluation. To mitigate bias, raters underwent comprehensive TPS training. The debriefings were analyzed through the DE-CODE framework. We utilized paired t-tests to examine performance improvements and linear regressions to assess the impact of reflective statements on performance, moderated by psychological safety.

**Results:**

Team performance significantly improved from the first to the second induction (*t* (9) = −2.512, *p* = 0.033). Senior physicians’ (*n* = 8) reflective statements predicted post-assessment TPS scores (*R*^2^ = 0.732, *p* = 0.061), while consultants (*n* = 7) and nurse anesthetists (*n* = 10) did not. Interaction analysis revealed no moderation effects, but a main effect indicated the significance of senior physicians’ reflective statements.

**Conclusion:**

This pilot study confirms the efficacy of the evidence-based debriefing app in enhancing anesthesia team performance. Senior physicians’ reflective statements positively influenced performance; however, no moderation effects were observed. The study highlights the potential of debriefing apps to streamline and enhance team debriefing processes, with significant implications for improving clinical practice and patient safety. Further research is needed to validate these findings on a larger scale and optimize the integration of debriefing into routine clinical practice.

## Background

Healthcare debriefings have the potential to enhance team learning and team performance in *ad hoc* teams. They reduce errors and improve patient safety (1, 2). It is a guided conversation among clinicians that aims to explore and understand the relationships among events, actions, thought and feeling processes, and performance outcomes of a clinical situation (3–7). A core element of debriefings is promoting experiential learning and thus reflecting/shared reflection which in turn may allow the development of strategies that can be applied in future performance episodes (8–12). Kolb’s Experiential Learning Theory posits that learning is a process where knowledge is created through the transformation of experience, following a cyclical model comprising four stages: Concrete Experience, Reflective Observation, Abstract Conceptualization, and Active Experimentation. This theory emphasizes that effective learning involves actively engaging in experiences, reflecting on them, conceptualizing the insights gained, and then applying these insights in practice (13). Debriefings are likely to be a suitable learning infra-structure (14, 15), particularly for *ad hoc* teams in healthcare with their temporal instability (16). While the potential of debriefings is increasingly recognized (17, 18) and empirical studies have demonstrated their benefits (19–21), they are still underutilized (15, 22, 23). Research has demonstrated that debriefings are only seemingly easy to conduct. In fact, they require a number of challenging conversational skills (24) and knowledge about team functioning (3, 21) which may be discouraging and requires the exploration of ways to help start and conduct debriefings (17). Also, research on organizational behavior suggests that many assumptions exist that may prevent healthcare personnel from engaging in debriefings. The so called “debriefing myths” include debriefing only when disaster strikes, debriefing is a luxury, senior clinicians should determine debriefing content, and debriefers must be neutral and nonjudgmental (1). These myths offer valuable insights into why current debriefing practices are *ad hoc* and not embedded into daily unit practices (1).

Different tools for conducting debriefings in the clinical setting exist for either hot [immediately after an event (4)] or cold [delayed hours to weeks after an event (4)] debriefings. These tools have in common that they have a similar framework and structure (5) but all of them lack a systematic analysis of the interaction between debriefers and participants namely how actions of debriefers relate to actions of participants; they mostly do not illuminate the debriefing process nor do they focus on specific questions to trigger participants’ double loop learning.

We therefore aimed to develop an evidence-based dynamic debriefing tool that contains evidence for the immediate effectiveness of selected debriefing and participant communications (6).

We have also tried to address the assumptions about debriefings mentioned above and why debriefings are rarely performed in the clinical setting. For example, the moderator of the debriefing is recommended, and participants are given a selection of topics to talk about in the debriefing (e.g., leadership, team coordination, speak up, team communication). In addition, participants are guided through the debriefing while using the debriefing app; for each phase of the debriefing, participants receive suggestions for effective question and they can also access current research results on selected crisis resource principles.

The objective of this observational pilot study was to test the impact of an evidence based debriefing app on anesthesia care providers’ performance. Based on team science and debriefing literature, we hypothesized that using the debriefing app in between two complex induction of anesthesia will enable team members to reflect and thus improve the performance of the second induction. Specifically, we tested the following hypotheses: (1) Team performance during anesthesia induction assessed by the Team Performance Scale (TPS) will increase after the debriefing and (2) the more reflective statements are verbalized during debriefings, the better the team performance is during the second induction for senior consultants, consultants, and registered anesthesia nurses, respectively. This relationship is moderated by psychological safety. Reflective statements were assessed via behavior observation and *in situ* behavior coding rather than relying on self-reports (7–9).

## Methods

The respective ethics committee determined this study to be exempt KEK-ZH-Nr. 2013-0592.

### Study design and inclusion/exclusion criteria

Data collection for this study took place at a central care-providing hospital in Switzerland. The participants included 10 male and 12 female anesthesia care providers. We observed participants performing complex inductions of general anesthesia in teams of 2 or 3. After the first induction, the participants used an app to debrief themselves, followed by a second complex induction of general anesthesia, which we observed again. Inductions were performed in the anesthesia induction room adjacent to the theater. Debriefings were conducted in a separate room immediately after the induction, facilitated by another anesthesia team that relieved the original team for this purpose.

Participants were recruited over 5 months for anesthesia in thoracic, visceral, vascular, or neurosurgery. Inclusion criteria included patients with an ASA classification of two or higher, requiring a central venous catheter, arterial catheter, thoracic epidural catheter, or double lumen tube, and complex patient positioning (e.g., prone or side position) (10). The exclusion criteria were anesthesia inductions in patients with an ASA classification 1 and 2, without extended monitoring or complex positioning. The anesthesia inductions included general anesthesia with and without thoracic epidural anesthesia, and all cases were elective surgical procedures. The teams consisted of one anesthesia consultant, one registrar, and one registered anesthesia nurse.

The anesthesia inductions took place in a designated induction room. After the placement of a thoracic epidural catheter, the usual steps such as preoxygenation, pharmacological induction, and pharmacological stabilization of blood pressure (within the usual range) were carried out.

### Data collection

Data were collected anonymously. Participants were informed about the study both verbally and through written documents, and written informed consent was obtained. Patient characteristics (age, physical status, ASA classification), type of surgical procedure, monitoring, duration of anesthesia induction, intubation method, and patient positioning were extracted from the patient file and anesthesia protocol. The debriefings were videotaped.

During inductions of anesthesia, team members were observed and assessed using the Team Performance Scale (TPS). The TPS analyzes the roles and responsibilities of team members and focuses on effective communication (11).

The TPS has been used as surrogate for the quality of the anesthesia induction. Raters were consultant anesthesiologists and anesthesia nurses with years of professional experience. All raters participated a two-hour rater training. The training included general information about the study purpose, a structured introduction into the rating systems and the observation method and rating of one videotaped induction of anesthesia using TPS under the direct guidance. To assess interrater reliability, two additional videotaped anesthesia inductions were evaluated. Training was considered complete if agreement between trainees and expert coders (Intraclass Correlation Coefficient) was.70 for both instruments indicating good interrater reliability (12). During anesthesia induction, the raters were placed closely to the anesthesia team and used TPS in real-time with direct observation.

The observation started with administering the first drug and ended with the handover to the surgical staff (25).

**Figure 1:**
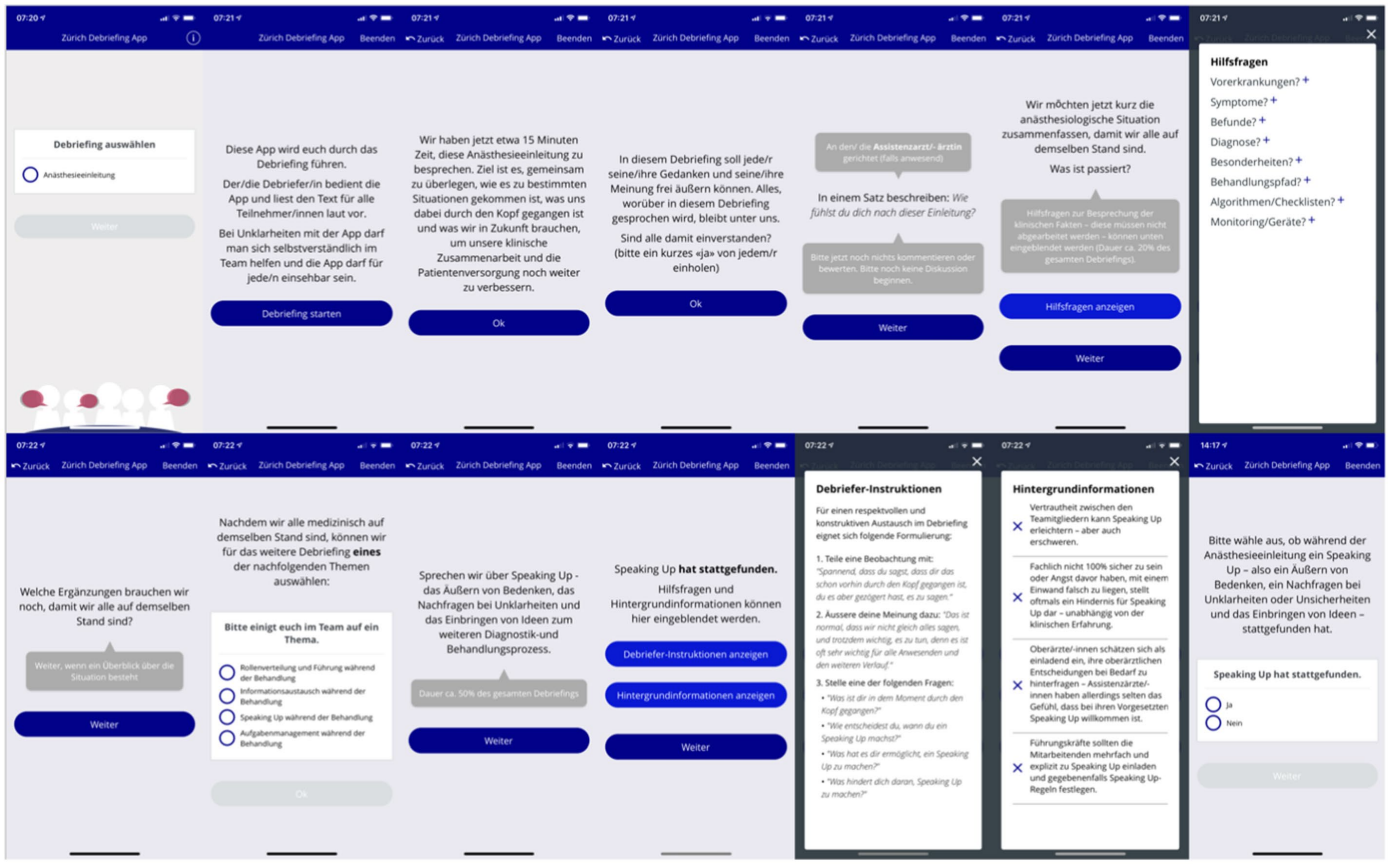
Screenshot of the application, showing the process of questioning.

**Figure 2:**
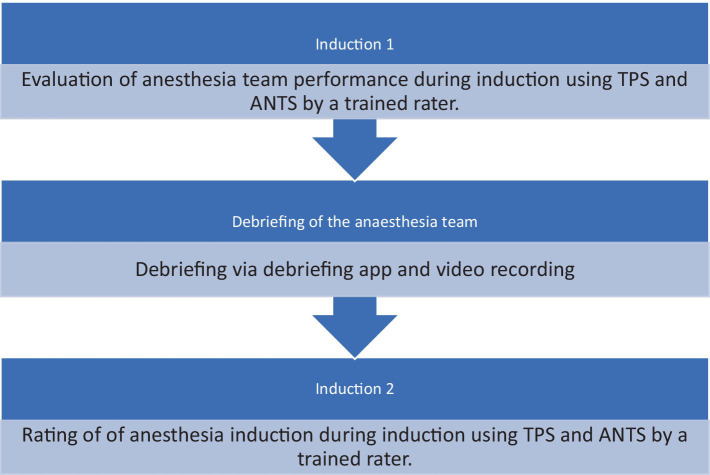
Study process.

After the first anesthesia induction was finished, the participants used the Zurich Debriefing App for a videotaped debriefing. Afterwards participants performed another induction of general anesthesia and underwent the same procedure. Both anesthesia inductions have been rated by different raters to avoid any biases (Figures 1, 2).

### Measurements

Participants completed a questionnaire after each debriefing. Psychological safety was measured using a validated German translation (26, 27) of the Team Psychological Safety scale.

### Data analysis

The debriefings have been observed remotely by the study team (7). In particular, they applied four codes of the DE-CODE, a valid and reliable coding scheme for assessing debriefers’ and learners’ communication in debriefings (28, 29). The authors focused on learners reflective statements/marker including learners analyses why something happened (DE-CODE: *description*), mentioning mental models (DE-CODE: *mental models*), learners conclusions about lessons learned or other actions that s/he could have done (DE-CODE: *conclusion*) as well as future-oriented action plans (DE-CODE: *action plan*). The anesthesia teams have been observed from the beginning of the debriefing until the end of the debriefing and reflective statements/marker have been recorded.

Behavioral coding was conducted using a standard personal computer and Excel sheet. To ensure interrater reliability, two coders independently coded 20% (36 out of 180) of the videotaped debriefings.

### Statistical analysis

Interrater reliability was assessed using the Intraclass Correlation Coefficient (ICC), suitable for ordinal, interval, and ratio scales. ICC values below 0.40 indicate poor reliability, between 0.40 and 0.59 are considered fair, 0.60–0.74 are good, and above 0.75 are excellent (30). Statistical analyses were performed using IBM SPSS V.26 software.

To evaluate the hypothesis that teams perform better during the second anesthesia induction, paired sample *t*-tests were conducted.

For the hypothesis concerning the relationship between the verbalization of reflective statements during debriefings and team performance during the second induction, linear regressions were performed. Additionally, a moderation analysis was conducted to examine whether this relationship is moderated by psychological safety.

## Results

### Interrater reliability

The ICC between two independent coders assessing 20% of the debriefings was 0.73, indicating good interrater reliability.

### Participants and descriptive data

Debriefings involved a minimum of two and a maximum of three participants, including attending physicians, resident physicians, and nurses with varying levels of experience in anesthesia. The mean duration of debriefings was 12.5 min, with a range of reflective statements made by participants. The average anesthesia experience was 9.17 years, on average; the team size for induction was 2.5 people. One person had never had simulation training with debriefing until then; all other participants were familiar with debriefing through simulation training.

### Descriptive data for inductions

Patients undergoing anesthesia inductions had an average ASA score of 3.05, with procedures primarily neurosurgical or thoracic in nature.

### Hypothesis testing

Results from paired sample *t*-tests revealed a significant increase in team performance from the first to the second anesthesia induction (*p* = 0.033), confirming the first hypothesis.

Regarding the second hypothesis, linear regression analyses showed that senior consultants’ reflective statements predicted post-assessment team performance scores (*R*^2^ = 0.732, *p* = 0.061), while consultants’ and registered anesthesia nurses’ statements did not significantly predict team performance. Moderation analysis did not reveal significant interactions between reflective statements and psychological safety for any group of anesthesia care providers. Therefore, the first hypothesis was confirmed, while the second hypothesis was partially supported, and the moderation hypothesis was not confirmed (Table 1).

**Table 1:** ANTS and TPS score.

Performance	ANTS	TPS	Total
Induction 1	3.43	4.27	7.70
Induction 2	3.81	4.66	8.46
Increase (%)	9.46	7.83	8.56

## Discussion

Aim of this pilot study was to test the impact of evidence based, guided debriefing app on anesthesia care providers’ team performance. Based on team science and debriefing literature, we hypothesized that using the debriefing app in between two complex induction of anesthesia will enable team members to reflect and thus improve the performance of the second induction. Specifically, we hypothesized that team performance will increase from first to second induction of anesthesia and that the more reflective statements are verbalized during debriefings, the better the team performance is during the second induction. In addition, we hypothesized that this relationship is moderated by psychological safety. We assessed reflective statements via behavior observation and team performance was assessed by using TPS. Results showed that our first hypothesis is confirmed.

Interpreting effect sizes is a critical aspect of research methodology. Cohen’s benchmarks (1988) classify effect sizes as small (*d* = 0.2), medium (*d* = 0.5), and large (*d* = 0.8), but their application should not be overly rigid. Despite these benchmarks, small effect sizes can hold significant practical implications, as seen in instances like interventions leading to a substantial reduction in suicide rates with an effect size of *d* = 0.1. While Cohen’s d for between-subject designs can be interpreted as a fraction of the standard deviation, offering a tangible measure, the most meaningful interpretation involves contextualizing the effect within existing literature and elucidating its practical implications. However, there is a lack of clear guidelines on how to undertake this process. Therefore, researchers must exercise discretion in interpreting effect sizes, considering both statistical benchmarks and the broader context of the research field (31).

Teamwork and thus patient safety can be improved by reflexivity, through reflexivity in debriefing, but also in a briefing or during action (32, 33). Based on this information, reflexivity in debriefing should be promoted.

In our study, the second hypothesis was that increased reflexivity in debriefing would lead to an improvement in TPS in the second induction, this was shown to be only partially significant. This was only shown in relation to the reflexivity of the senior doctors’ statements. However, this was probably also due to the small sample in the pilot study. This would have to be analysed again in a larger study and especially the participants’ share of conversation in the debriefing as well as the reflection markers would have to be considered further.

The second hypothesis is only confirmed for senior consultants, a main effect is shown in the reflective statements of the senior consultants and an increased performance post, otherwise no moderation effects were shown. The results show that our second hypothesis is not confirmed.

The strengths of the study are certainly demonstrated by the ease of conducting the debriefing using an app on a smartphone or pad, as this can be done in a resource-efficient and simple way. After all, the use of smartphones in everyday clinical practice is now well accepted by most doctors and nurses (34). Through the app, the team can be guided neutrally through the debriefing and the participants are tempted to reflect on their actions in the team. The limitations of this study are that it is a single center study and has only a small number of cases. Furthermore, organizing the same team for two consecutive complex anesthesia inductions proved to be a challenge.

It is noteworthy to highlight our adherence to recommendations put forth, as evidenced by the alignment of our approach with the findings elucidated in the systematic review on clinical debriefing tools: attributes and evidence for use. Additionally, our reference to authoritative documents such as Healthcare Simulation Standards of Best Practicetm, The Debriefing Process, Reflective debrief and the social space: offload, refuel, and stay on course, and Clinical debriefing: TALK© to learn and improve together in healthcare environments, underscores the robust methodology.

The incorporation of reflexivity during debriefing sessions has been shown in contemporary literature to be conducive to enhancing teamwork dynamics and bolstering patient safety measures (35). This is particularly pertinent given the complexities inherent in healthcare environments. Furthermore, our findings pertaining to the second hypothesis, while partially significant, warrant nuanced interpretation. The observed partial significance could be attributed, in part, to the relatively modest sample size utilized in our study. Moving forward, it may be prudent to delve deeper into the conversational dynamics within debriefings, potentially shedding light on the need to ensure equitable participation beyond senior consultants. It is plausible that other anesthesia providers may have contributed disproportionately to the overall discourse. Consequently, future analyses should prioritize assessing the balance of reflective markers rather than focusing solely on individual contributors.

The accessibility and dynamic nature of our debriefing application are notable, serving as an effective tool in guiding users through the debriefing process. By reducing barriers, such as complexity and time constraints, our application streamlines the debriefing experience, making it more accessible and resource-efficient in clinical settings.

Moreover, our findings underscore the versatility of our approach, as it is suitable for both hot and cold debriefings, as advocated by Sugarman (5). However, it is imperative to acknowledge the limitations inherent in our study design. As a single-center study with a modest sample size, our findings may not be generalizable to broader contexts. Furthermore, the pilot nature of our study posed challenges in ensuring stable team compositions for two sequential inductions, potentially impacting the robustness of our findings. Additionally, our study focused exclusively on a single discipline within healthcare, further limiting the generalizability of our findings.

Finally, despite concerns surrounding the integration of smartphone applications in clinical practice, our findings indicate a prevailing positive attitude among healthcare professionals toward their use. This trend is supported by the burgeoning adoption of smartphones among healthcare professionals over the past decade, with approximately 80% of doctors and 85% of medical trainees utilizing smartphones in their professional capacities (34).

For clinicians, these findings present significant advantages. They allow for systematic and structured debriefings to be conducted without a loss of time. Additionally, they document the learning effect. Furthermore, team members are trained to independently conduct effective debriefings. Based on the findings, the use of the application can be recommended; however, the effect of the subject of debriefing should not be overlooked (36). In this study, only non-critical situations were discussed, aligning with the Safety II concept by Hollnagel et al. (37). Whether this structure yields similarly positive effects in situations involving incidents remains to be seen.

We hope that this pilot study will help to confirm our hypotheses in a larger study and create a tool through this app that can better integrate debriefing into everyday clinical practice and thus improve team performance and patient safety.

## Data availability statement

The raw data supporting the conclusions of this article will be made available by the authors, without undue reservation.

## Ethics statement

Ethical review and approval was not required for the study on human participants in accordance with the local legislation and institutional requirements. Written informed consent from the patients/participants was not required to participate in this study in accordance with the national legislation and the institutional requirements.

## Author contributions

CS: Conceptualization, Data curation, Formal analysis, Funding acquisition, Investigation, Methodology, Project administration, Resources, Software, Supervision, Validation, Visualization, Writing – original draft. JS: Data curation, Project administration, Visualization, Writing – original draft. MK: Conceptualization, Data curation, Formal analysis, Funding acquisition, Methodology, Project administration, Resources, Supervision, Writing – original draft. BG: Conceptualization, Funding acquisition, Methodology, Resources, Supervision, Validation, Writing – original draft, Writing – review & editing.
